# The German version of the neurofibromatosis 2 impact on quality of life questionnaire correlates with severity of depression and physician-reported disease severity

**DOI:** 10.1186/s13023-022-02607-z

**Published:** 2023-01-06

**Authors:** Anna Cecilia Lawson McLean, Anna Freier, Aaron Lawson McLean, Johannes Kruse, Steffen Rosahl

**Affiliations:** 1 Department of Neurosurgery, Helios Klinikum Erfurt, Nordhäuser Str. 74, 99089 Erfurt, Germany; 2 Department of Psychosomatics and Psychotherapy, University of Giessen, Giessen, Germany; 3 Department of Neurosurgery, University Hospital Jena, Jena, Germany

**Keywords:** Neurofibromatosis 2, Quality of life, Depression, Disease severity

## Abstract

**Background:**

Neurofibromatosis type 2 (NF2) is a rare genetic disease that causes a wide range of disabilities leading to compromised quality of life (QOL). There is clear need for a validated disease-specific tool to assess quality of life among German-speaking patients with neurofibromatosis type 2 (NF2). The NFTI-QOL questionnaire has produced useful results in English-speaking cohorts. The aim of this study was to produce and validate a German version of the NFTI-QOL (NFTI-QOL-D) and to correlate QOL scores with a depression score (PHQ-9) and clinical disease severity.

**Methods:**

The original English-language NFTI-QOL was translated into German and then back-translated in order to preserve the questionnaire’s original concepts and intentions. A link to an online survey encompassing the NFTI-QOL-D and the PHQ-9 depression questionnaire was then sent to 97 patients with NF2 by email. The respondents’ scores were compared to clinician-reported disease severity scores.

**Results:**

77 patients completed the online survey in full. Internal reliability among NFTI-QOL-D responses was strong (Cronbach’s alpha: 0.74). Both PHQ-9 and clinician disease severity scores correlated with NFTI-QOL-D scores (Pearson’s rho 0.63 and 0.62, respectively).

**Conclusions:**

The NFTI-QOL-D is a reliable and useful tool to assess patient-reported QOL in German-speaking patients with NF2. The correlation of QOL with both psychological and physical disease parameters underlines the importance of individualized interdisciplinary patient care for NF2 patients, with attention paid to mental well-being as well as to somatic disease manifestations.

## Background

Over the past decades, there has been a paradigm shift from focussing solely on objective and clinician-assessed outcomes such as overall survival or progression-free survival to patient-reported outcomes such as quality of life. It is increasingly accepted that a clinicians’ focus should be on the patients’ ability to function in daily activities and their self-perceived quality of life. This is especially true for patients with chronic diseases such as neurofibromatosis type 2 (NF2), which is a rare autosomal-dominant hereditary disease. Patients with NF2 develop widespread tumours of the central and peripheral nervous systems such as vestibular schwannomas, meningiomas, spinal ependymomas or peripheral nerve schwannomas. In addition, polyneuropathy and cataract are commonly associated with NF2 [1]. This in turn leads to a wide range of disabilities, including hearing loss, motor and sensory deficits.

In 2012, Hornigold et al. introduced the English-language disease-specific neurofibromatosis 2 impact on quality of life (NFTI-QOL) questionnaire and demonstrated its usefulness in a large multicentric NF2 patient cohort in the UK. The NFTI-QOL questionnaire showed strong correlation with the SF-36 and EuroQOL questionnaires, which are both well-established and non-disease-specific tools for the assessment of quality of life [2].

The advantage of a disease-specific questionnaire, however, is the targeted evaluation of disease aspects that are relevant to NF2 patients and the omission of generic or superfluous items. The more pointed (and shorter) the questionnaire the more likely patients are to complete it and the easier it is to interpret the results. This is especially true for multi-morbid patients with visual and manual disabilities as in NF2. The NFTI-QOL is shorter than generic QOL questionnaires such as the SF-36 and can be completed in approximately 3 min [2].

Further studies in UK, American and Canadian contexts yielded similar results to the original study and found correlations between quality of life and patient- and physician-reported disease severity [3–5]. However, there was no association between the size of the pathognomonic tumours of NF2, vestibular schwannomas, and patient-reported quality of life [3].

There is a large German-speaking group of NF2 patients, which has not yet been systematically assessed with respect to disease-specific quality of life. Based on the reported incidence of 1 in 25,000–40,000 [6, 7] and the current German population of 83.2 million [8], there are estimated to be over 2,000 German NF2 patients, with even more native German-speaking NF2 patients in Austria and Switzerland. From this large number of German-speaking patients much could be learned about improving disease-related health outcomes and quality of life in NF2, as well as the lived experience of those with NF2.

In addition, the impact of healthcare provision, encompassing the structures and funding of NF2 care at regional and national level, in relation to NF2 outcomes including quality of life, has not been addressed. A standardised quality of life instrument could facilitate international comparison of patient-reported outcomes and enhance the validity and reproducibility of comparisons across centres as well as longitudinal follow-up of individual patients.

The aim of this study was to design and implement a standardised German version of the English-language NFTI-QOL and to assess quality of life in a large German-speaking cohort of NF2 patients.

## Methods

### Translation of the NFTI-QOL into German

*Forward translation* As a first step, the original English version of the NFTI-QOL questionnaire was translated into German by ACLM (a native German speaker with profound knowledge of the English language who lives in the target country). The aim was to produce a version which was semantically and conceptually as close as possible to the original questionnaire.

*Back translation* The German version was then back-translated into English by ALM (a native English speaker with profound knowledge of the German language who lives in Germany but spent most of his life in the UK).

**Fig. 1:**
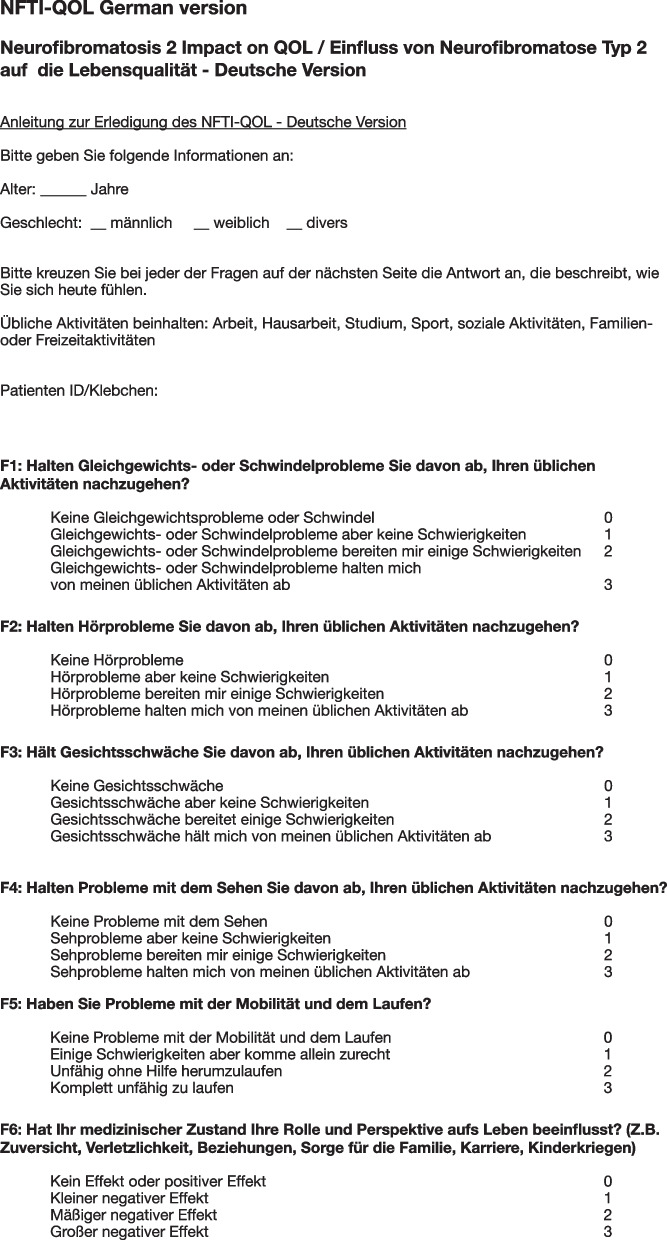

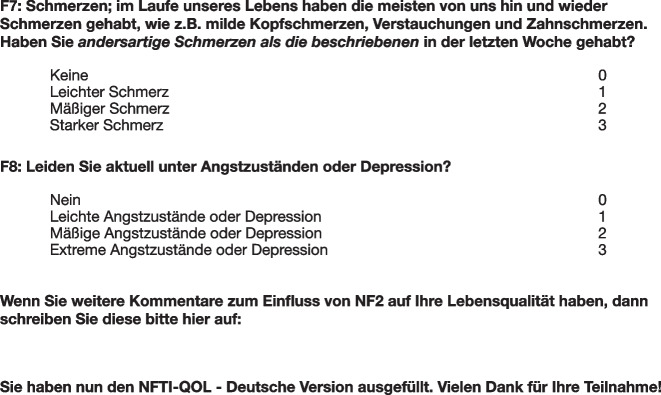
NFTI-QOL German version. Like the original version the questionnaire consists of 8 items with 4 possible answers each. Depending on symptom severity, 0 to 3 points are granted per category, resulting in up to 24 points in total

*Harmonisation* Any discrepancies with the original English version were discussed by ACLM and ALM until agreement was reached. AF (a native German speaker who lives in Germany) consecutively assessed the generated German version of the NFTI-QOL (henceforth “NFTI-QOL-D”) for intelligibility and readability. The finalised NFTI-QOL-D can be found in Fig. 1.

### Online survey

An online survey was created including the NFTI-QOL-D and the German version of the PHQ-9 questionnaire (9-item patient health questionnaire, a tool for the assessment of depression) [9, 10]. The link to the online survey was sent to 97 German-speaking patients with NF2 treated at the supraregional Erfurt Neurofibromatosis Centre, representing all NF2 patients treated at the centre for whom email addresses were available.

### Utilised questionnaires and scores

*NFTI-QOL* This disease-specific questionnaire consists of 8 items relating to different aspects of quality of life with 4 possible answers each. Depending on symptom severity, 0 to 3 points are granted per category, resulting in up to 24 points in total. The higher the score, the lower the quality of life. Hornigold et al. found no significant correlation with age or gender (ρ = 0.246 and ρ = 0.025, respectively). It showed good internal reliability (α = 0.87) and a good ability to detect significant longitudinal changes in the quality of life of individuals. The free text section, which was included in the original version of the NFTI-QoL was not mentioned in our online survey as it was already stated by Hornigold et al. that the free text section did not yield any additional information and patients rather affirmed the importance of the items included in the questionnaire [2].

*PHQ-9* A commonly used questionnaire with 9 items which each assess one of the 9 DSM IV criteria for depression. Depending on symptom severity, 0 to 3 points are granted per category, resulting in up to 27 points in total. The higher the score, the higher the likelihood of clinically relevant depression. It has been widely validated and is considered the most reliable screening tool for depression [11–13].

**Fig. 2:**
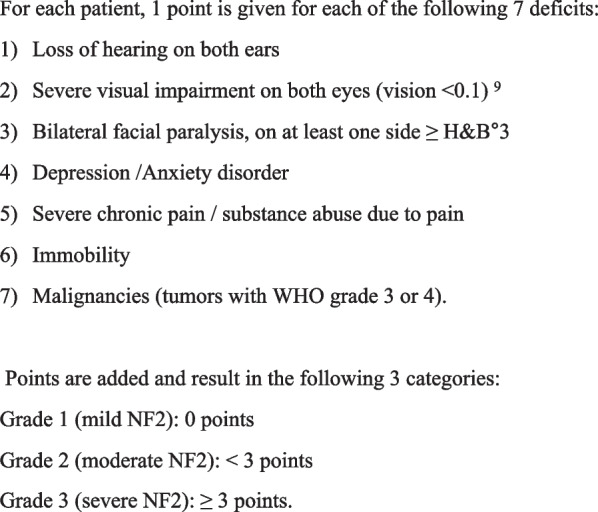
Disease severity score

*Disease severity score* The utilised clinician-reported disease severity score is based on the presence or absence of 7 symptoms with major impact on the patient’s life. Per symptom 1 point is added and based on the total sum disease severity is divided into 3 levels: mild disease, moderate disease and severe disease (Fig. 2 [1]). This score was validated for internal consistency and correlation with clinical parameters, such as number of hospitalisations per year. The disease severity score was calculated for each patient based on medical records of the patients’ last admissions or out-patient presentations.

### Ethics approval

The study was approved by the local ethics committee and informed consent was obtained from all participating patients.

### Statistics

NFTI-QOL-D scores were calculated and checked for internal consistency and correlation with patient-specific depression and disease severity scores. R Studio version 2022.02.3 was utilised for descriptive statistics, Cronbach’s α and Pearson’s correlation analyses. Median numbers were calculated due to the number of participants and expected non-normal or near-normal distribution.

## Results

77 patients (49 female, 28 male) completed the online survey in full. This equates to a return rate of 79%. The median age was 38 years (range 16–68 years).

**Fig. 3:**
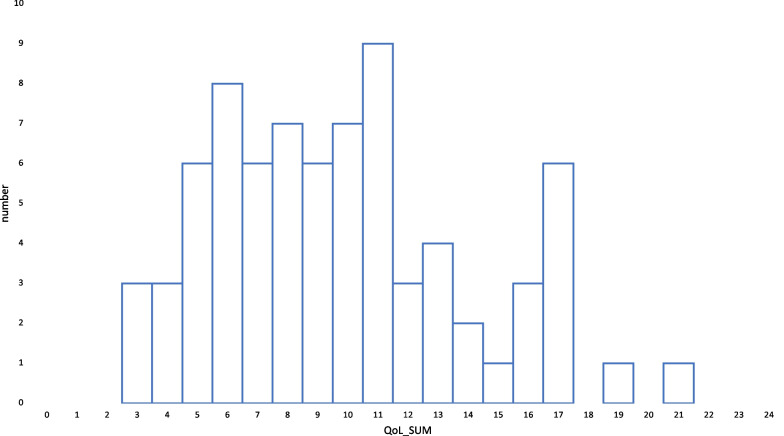
Results of NFTI-QOL German version from 77 patients. The median overall score was 9. There was a nearly normal distribution of all scores

The median overall score for NFTI-QOL-D ± SD was 9 ± 4.2 with a nearly Gaussian distribution (Fig. 3). There was good internal reliability (Cronbach’s alpha 0.74, 95% CI 0.61–0.81). For scores of individual items please refer to Table 1.

**Table 1:** Results of NFTI-QOL-D including scores of individual items

	Vertigo	Hearing	Paresis	Vision	Mobility	Outlook	Pain	Depression/Anxiety	SUM
MEDIAN	1	2	1	1	1	2	1	1	9
SD	0.97	0.87	0.91	0.86	0.69	0.95	1.01	0.93	4.2

Individual median scores ± standard deviation (SD) for each item of the NFTI-QOL-D questionnaire are shown. The median overall score for NFTI-QOL-D ± SD was 9 ± 4.2 with a nearly Gaussian distribution

The median overall score ± SD for the PHQ-9 questionnaire was 7 ± 5.4. There was a strong correlation between NFTI-QOL-D and PHQ-9 (Pearson’s rho = 0.63, 95% CI 0.48–0.75). There was a moderate correlation between the item assessing anxiety and depression within the NFTI-QOL-D and the PHQ-9 (Pearson’s rho = 0.57, 95% CI 0.40–0.71).

**Fig. 4:**
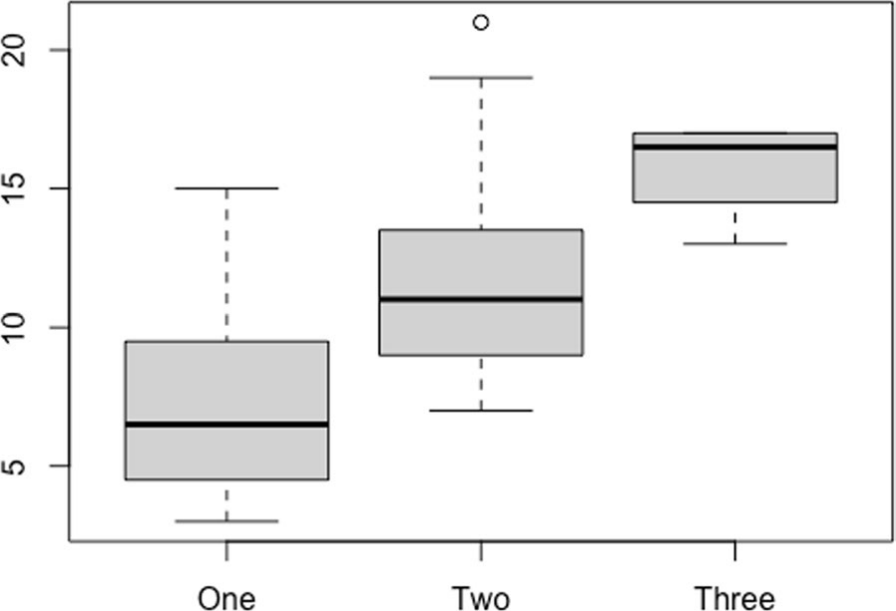
There was a strong correlation between NFTI-QOL-D and clinician-reported disease severity (Pearson’s rho = 0.62, 95% CI 0.42–0.76). The boxplot shows the median NFTI-QOL-D scores by physician-rated disease severity score on the x-axis

The 3-level disease severity score was available for 55 of the 77 patients who completed the questionnaire. 24/55 patients were grouped into level 1 (mild disease), 27/55 were grouped into level 2 (moderate disease), and 4/55 patients were grouped into level 3 (severe disease). There was a strong correlation between NFTI-QOL-D and clinician-reported disease severity (Pearson’s rho = 0.62, 95% CI 0.42–0.76; Fig. 4).

There was no statistical difference between median overall scores of the NFTI-QOL-D and the PHQ-9 between the 55 patients personally known to our institution and the 22 unknown patients (*p* > 0.5 in Wilcoxon rank sum test).

26 of the 55 patients known at our institution were currently under bevacizumab treatment. The overall NFTI-QOL-D scores for patients with bevacizumab and those without were 10 ± 3.9 and 9 ± 4.9, respectively. There was no statistically significant difference (*p* > 0.5 in Wilcoxon rank sum test).

## Discussion

There was a high response rate of the online survey (77 responses to 97 invitations). The average return rate for online surveys is 46–51% [14]. The high response rate in our study may reflect high motivation amongst the NF2 community. The fact that 22 of the completed forms could not be attributed to patients known to staff at the Erfurt Neurofibromatosis Centre suggests that contacted NF2 patients forwarded on the survey to acquaintances and family members who are also affected by the disease.

The median overall score of the NFTI-QOL found in this German cohort correlates with previous findings in English-speaking patient collectives [2–4]. Internal reliability was similar to that of the original NFTI-QOL questionnaire (0.74 vs. 0.87) [2]. We therefore consider the NFTI-QOL-D as reliable and conclusive as the original version.

We found strong correlation between the NFTI-QOL-D and the PHQ-9, indicating that there is a connection between quality of life and depression severity. Interestingly, the correlation between the item assessing depression and anxiety within the NFTI-QOL-D and the PHQ-9 was lower than between the overall values. This underlines the importance of the usage of specific tools for different aspects of patient care. In addition, psychological assessment is recommended in all patients with neurofibromatosis type 2 as depression and anxiety are common in this patient collective. Psychological care should form a central pillar of the interdisciplinary management of these highly complex patients.

There was also a strong correlation between quality of life and clinician-reported disease severity. These results accord with findings from English-speaking cohorts, despite the use of a variety of disease severity scores [2, 3]. There was a slightly better correlation between the NFTI-QOL-D and physician-rated disease severity in our cohort than in the cohort presented by Hornigold et al. and their implemented clinician-rated severity score (Pearson’s rho = 0.62 vs. rho = 0.51) [2]. A validated and reliable tool for physician-reported assessment of disease severity would be desirable for future research and standardised follow-up investigations.

The NFTI-QOL-D is an age-independent tool for adults. There is a wide clinical spectrum of NF2 and disease severity does not necessarily correlate with age. However, on an individual basis, the disease might progress, which may affect QOL in a negative way. On the other hand, surgical, medical or psychological interventions may have a positive effect on symptom severity or resilience which then leads to an improvement of QOL. Therefore, we consider an annual evaluation (alongside the patients’ routine clinical and MRI follow-up investigations) with the proposed NFTI-QOL-D questionnaire useful. Paper and online options should be provided to account for individual preferences and abilities.

This study underlines the importance of interdisciplinary care for patients with NF2 in specialised centres. Physicians and surgeons who work at these centres should not only be experts in their respective fields but should also be experienced in dealing with chronically ill patients. In addition, many patients with NF2 require psychological treatment. However, psychological therapy may be challenging as loss of hearing and vision are common. Therefore, psychologists with neurooncological training and sign language skills are urgently needed to provide adequate care for these patients.

## Conclusions

The NFTI-QOL-D is a reliable and useful tool to assess patient-reported quality of life in German-speaking patients with NF2. We recommend the implementation of this validated questionnaire in further studies in order to guarantee reproducibility across centres and contexts.

There was a strong correlation between quality of life and both psychological and physical-functional factors. This underlines the importance of a well-coordinated, interdisciplinary, holistic management of NF2 patients in specialised centres, where mental wellbeing as well as somatic disease manifestations are individually addressed.

## Data Availability

The datasets used and/or analysed during the current study are available from the corresponding author on reasonable request.

## Abbreviations

DSM IV: Diagnostic and Statistical Manual of Mental Disorders Version IV
MRI: Magnetic resonance imaging
NF2: Neurofibromatosis type 2
NFTI-QOL: Neurofibromatosis 2 impact on quality of life questionnaire
NFTI-QOL-D: German version of the neurofibromatosis 2 impact on quality of life questionnaire
PHQ-9: Patient health questionnaire including 9 items
QOL: Quality of life
SD: Standard deviation
SF-36: Short Form 36 questionnaire
